# Integration options for high energy efficiency and improved economics in a wood-to-ethanol process

**DOI:** 10.1186/1754-6834-1-4

**Published:** 2008-04-15

**Authors:** Per Sassner, Guido Zacchi

**Affiliations:** 1 Department of Chemical Engineering, Lund University, P.O. Box 124, SE-221 00 Lund, Sweden

## Abstract

**Background:**

There is currently a steady increase in the use of wood-based fuels for heat and power production in Sweden. A major proportion of these fuels could serve as feedstock for ethanol production. In this study various options for the utilization of the solid residue formed during ethanol production from spruce, such as the production of pellets, electricity and heat for district heating, were compared in terms of overall energy efficiency and production cost. The effects of changes in the process performance, such as variations in the ethanol yield and/or the energy demand, were also studied. The process was based on SO_2_-catalysed steam pretreatment, which was followed by simultaneous saccharification and fermentation. A model including all the major process steps was implemented in the commercial flow-sheeting program Aspen Plus, the model input was based on data recently obtained on lab scale or in a process development unit.

**Results:**

For the five base case scenarios presented in the paper the overall energy efficiency ranged from 53 to 92%, based on the lower heating values, and a minimum ethanol selling price from 3.87 to 4.73 Swedish kronor per litre (0.41–0.50 EUR/L); however, ethanol production was performed in essentially the same way in each base case scenario. (Highly realistic) improvements in the ethanol yield and reductions in the energy demand resulted in significantly lower production costs for all scenarios.

**Conclusion:**

Although ethanol was shown to be the main product, i.e. yielding the major part of the income, the co-product revenue had a considerable effect on the process economics and the importance of good utilization of the entire feedstock was clearly shown. With the assumed prices of the co-products, utilization of the excess solid residue for heat and power production was highly economically favourable. The study also showed that improvements in the ethanol yield and reductions in the energy demand resulted in significant production cost reductions almost independently of each other.

## Introduction

Since the introduction of electricity certificates in May 2003 there has been a steady increase in the use of bioenergy for heat and power production in Sweden. Wood-based fuels (forestry residues, bark, chips, pellets, etc.) are one of the main contributors to this increase. A major proportion of these fuels could serve as feedstock for ethanol production. Instead of burning the entire material, the carbohydrate fraction could be converted to ethanol and the lignin-rich solid residue could then be used as a solid fuel.

A process based on enzymatic hydrolysis and fermentation is currently regarded as the most promising option for the conversion of carbohydrates in lignocellulosic materials into ethanol in an energy-efficient way, with high yields and low production cost [1,2]. The complex structure of lignocellulosic materials, the presence of various hexose and pentose sugars in hemicellulose, and the presence of various compounds that inhibit the fermenting organism constitute physical barriers that add to the production cost, which makes full-scale introduction economically risky. Pilot-scale production plants and pre-commercial demonstration facilities have recently been brought into operation in several places world-wide [3-6]. However, the process concept has not yet been demonstrated on an industrial scale.

The live steam required in the ethanol process is generated by burning part of the solid residue (together with the concentrated liquid from evaporation of the stillage and possibly some biogas generated in waste water treatment). The excess solids can be utilized in a number of different ways, resulting in different co-products contributing to the overall revenue. The solid residue can be turned into pellets, which can be sold on the residential pellet market. Electricity can be generated and the excess can be sold to the grid, while waste heat can be provided to a district heating system. The latter option restricts the location of the plant as there must be a demand for the heat available.

Production of other chemicals besides ethanol from part of the carbohydrate or lignin fractions of lignocellulosic materials has been suggested as a way to improve process economics [7,8]. However, in most comprehensive techno-economic studies performed in recent years utilization of the lignocellulosic feedstock for co-production of ethanol and electricity or pellets has served as the basic process concept [9-12]. This study focuses on the utilization of the excess solids for energy purposes in ethanol production from spruce. Different scenarios, with the ethanol production process as a stand-alone plant, or integrated with either a pellet production facility or a combined heat and power plant, were compared in terms of overall energy efficiency and process economics. The effect of changes in the process performance, such as variations in the ethanol yield and/or the energy demand, was also studied.

The ethanol process was based on SO_2_-catalysed steam pretreatment followed by simultaneous saccharification and fermentation (SSF). Treatment of chipped biomass with high-pressure steam, especially with the addition of a small amount of an acid catalyst such as sulphuric acid or sulphur dioxide, has been shown to be a successful method of pretreating several lignocellulosic materials prior to enzymatic hydrolysis [13-19]. Compared with separate enzymatic hydrolysis and fermentation, SSF has been shown to be less capital intensive and to result in higher overall ethanol yields, which is economically highly beneficial [10,20,21].

An Aspen Plus model which included all the major process steps was used in the study, since changing the conditions in one process step is likely to affect other parts of the process. Process data for the pretreatment and SSF steps were based on experimental results recently obtained on lab scale or in a process development unit.

It should be emphasized that the results obtained in the techno-economic study should not be regarded as absolute values. A large number of assumptions based on experimental data, literature and rules of thumb lie behind the estimates of the production cost, energy demand, etc. The costs, in particular, should mainly be used for comparison of the various process alternatives within this study. Comparisons with costs reported in similar studies employing other underlying assumptions in the evaluations should be undertaken with great care.

## Materials and methods

### Raw material

In Sweden softwood is the dominant source of lignocellulosic material and in Swedish research spruce has been the most extensively studied material as feedstock in a wood-to-ethanol process [1,22-26]. The hemicellulose in spruce is mainly made up of mannose units, which are readily fermented by baker's yeast. The pentose fraction is low, thus moving the focus, to some extent, away from the problems associated with pentose fermentation. The dry raw material in the current study was assumed to consist of 59.3% hexosans (glucan, mannan and galactan), 8.0% pentosans (xylan and arabinan) and 27.5% lignin. The remaining 5.2% is made up of acetyl groups, ash and extractives. The dry matter (DM) content was assumed to be 45%. Theoretically, 426 litres of ethanol can be produced from the hexose sugars per dry metric ton of raw material. An additional 59 litres can be produced if the pentose sugars are also converted to ethanol.

### Process description

**Figure 1:**
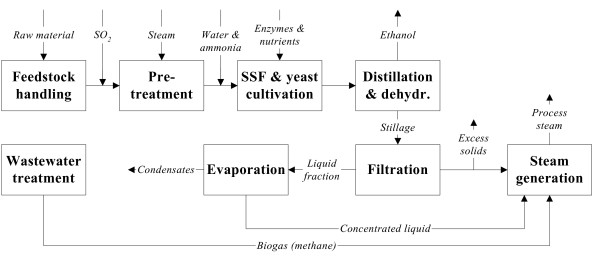
Overview of the proposed ethanol production process.

An overview of the assumed ethanol process is shown in Figure 1. The proposed ethanol plant is assumed to be located in Sweden, with the capacity to process 200 000 tons of dry raw material per year. It is run by 28 employees and the annual on-stream time was set at 8000 hours. Primary steam is assumed to be available at 20 and 4 bar, and secondary steam is used to replace live steam when possible. The process design has been described in detail elsewhere [11,26,27] and will only be briefly discussed here.

#### Ethanol production

The conversion of the carbohydrates is performed with SO_2_-catalysed steam pretreatment followed by SSF. Process data for the pretreatment (205°C, 5 minutes, 1.25% SO_2_) and SSF steps were based on results recently obtained in the experimental and analytical work performed on lab scale or in a process development unit at the Department of Chemical Engineering, Lund University, Sweden. After pretreatment, 63% of the original hexosan content remains in the solid phase and 28% is recovered as water-soluble sugars. The corresponding figures for the pentosan fraction are 10% and 37%. The total carbohydrate recovery after pretreatment is 87%. The dry weight loss due to the formation of volatile compounds is 9.9%.

Yeast is cultivated in aerated propagation tanks on sugars present in a liquid stream that is separated from the pre-treated slurry. SSF is performed at a water-insoluble solids (WIS) concentration of 10% with 2 g/L yeast and an enzyme dosage corresponding to 15 filter paper units (FPU) per gram WIS. Ammonia is used to neutralise the pretreated slurry. This is preferable to the addition of lime, which would increase the risk of fouling the heat exchanger surfaces throughout the ethanol plant [28,29], and to the use of sodium hydroxide, which would result in an increased amount of sodium salts in the combustion step and should, therefore, be avoided. During SSF glucan is hydrolysed and most of the hexose sugars (glucose, mannose and galactose) are fermented to ethanol, resulting in an SSF broth with 4.0% (w/w) ethanol. The conversion was set to match experimental data. The remaining sugars are converted to byproducts or remain unfermented. Furfural and 5-hydroxymethylfurfural are reduced to their corresponding alcohols. The overall ethanol yield, including ethanol losses in the process, and taking into consideration the fact that part of the sugars are used for yeast production, is 296 litres per metric ton dry feedstock. This corresponds to 69.4% of the theoretical value, based on the hexosan content in the raw material. (Additionally 22 litres ethanol would be produced per metric ton dry feedstock, if 90% of the pentose sugars present in SSF were converted to ethanol together with the hexoses.)

#### Recovery of ethanol, solids and water

Ethanol is concentrated to 99.8% by means of distillation, consisting of three thermally coupled columns, and molecular sieve adsorption. The first two distillation columns are stripper columns (25 trays each with 50% Murphree efficiency) working in parallel with top stage pressures of 3 and 1.25 bar. The distillate streams are sent to a rectification column (45 trays with 75% Murphree efficiency), which is operated at a top stage pressure of 0.3 bar.

The solid phase of the stillage streams leaving the stripper columns is separated in a filter press resulting in a solid residue containing 40% WIS. The liquid phase is concentrated to 60% DM in an evaporation system consisting of five evaporator effects in a forward-feed arrangement. Steam at a pressure of 4 bar is used as heating medium in the first effect and the condenser operates at 0.2 bar. Boiling point elevation was accounted for [30] and overall heat transfer coefficients were varied between 600 and 2000 W/m^2^°C depending on the temperature and concentration of the liquid. Most of the evaporation condensates are recycled to the process to reduce the use of fresh water. Before this can take place, further purification was assumed to be necessary to avoid the accumulation of compounds with inhibitory effects on the yeast and enzymes. For this purpose, a stripper column (with the design based on the work of Olsson et al. [31]), equipped with all necessary auxiliary equipment, was included in the economic evaluation. The concentrated evaporation liquid is sent to combustion.

A waste water treatment facility was also included in the model, in which the flash streams from pretreatment and part of the evaporation condensates are treated by anaerobic digestion, followed by an aerobic step. It should be emphasized that the performance of this step is very uncertain due to the lack of experimental data regarding the treatment of the kind of substrate obtained in a wood-based ethanol process and requires further investigation. It was assumed that 50% of the carbon oxygen demand (COD) is converted to biogas, producing 0.35 m^3 ^methane per kg COD consumed. The biogas is burnt to generate steam.

#### Pellet production

In the process configurations including pellet production (scenarios B and C, see below), drying of the solid residue is integrated with the ethanol process. The rest of the pellet production facility is only accounted for in the economic evaluation. The excess solid residue, i.e. the fraction of the solids not required for steam generation, is dried to 88% DM in a steam dryer working at 4 bar with superheated steam as the drying medium. The secondary steam that is generated is used to replace live steam in the ethanol process.

#### Generation of steam, electricity and heat for district heating

Superheated steam (91 bar, 470°C) is generated in the steam boiler (except in scenario B, see below) by burning the solid residue together with the concentrated liquid from evaporation and the biogas (methane) generated in the waste water treatment facility. The moisture content of the fuel is about 50% and the heat losses in the boiler were assumed to be 1%. The generated steam is allowed to expand to 0.75 bar through a turbine system, consisting of a high-pressure part (outlet pressure 4 bar) and a low-pressure part, to generate electricity. The isentropic efficiency of the turbines was set to 90 and 85% in the high-and low-pressure parts, respectively, and the mechanical and electrical efficiency of the generator was assumed to be 97%. Steam required in the ethanol process (20 and 4 bar) is withdrawn. Some steam is also used to preheat the feed water to the boiler. The pressure in the feed water tank is 4 bar and the temperature 140°C. The flue gases leaving the boiler are used to preheat the feed water to 220°C and the air used for combustion. The temperature of the flue gases after the air heater is fixed at 150°C and is maintained by varying the flow through the steam cycle.

The temperature of the return water from a district heating system is raised from 48 to 90°C by passing the stream through a flue gas condenser and the turbine condenser. The rather high moisture content of the fuel gives rise to a considerable amount of water vapour in the flue gases. Some of this latent heat is utilized in the flue gas condenser, in which the temperature of the flue gases is reduced to 50°C. The return temperature of 48°C and the outlet temperature of 90°C of the district heating stream are typical mean values [32]. In reality, these temperatures vary over the year. The implementation of the flue gas condenser increases the steam boiler efficiency from 91 to 110%, based on the lower heating value (LHV) of the fuel.

#### Suggested process configurations

In the current study, five scenarios, A-E, were considered. In all scenarios steam required for the ethanol process was generated by burning all (scenarios A, D and E) or part (scenarios B and C) of the solid residue together with the concentrated liquid from evaporation and the biogas (methane) generated by waste water treatment. A summary of the scenarios and products is given in Table 1. In scenario A the steam is allowed to expand to a pressure of 0.055 bar and cooling water is used to condense the steam leaving the turbine. In scenario B no steam turbines are included. Thus, no electricity is produced, and the electricity required to run the plant is purchased. The steam boiler is only used to generate steam for the ethanol process and excess solids are pelletized. In scenario C the steam boiler is supplemented with a back-pressure steam turbine (outlet pressure 4 bar) for the generation of electricity. Consequently, a larger fraction of the solid residue than in scenario B is required as fuel in the steam boiler. Scenario C does not necessarily result in excess electricity (see Results & Discussion). In scenarios D and E heat from the flue gas and turbine condensers is provided to a district heating system. In scenario E the pressure of the evaporation condenser is increased so that this heat can also be supplied to the district heating system. The evaporation system still consists of five effects, but the heat transfer area required is increased due to the smaller temperature differences, which adds to the capital cost.

**Table 1:** Products in the different scenarios

	**Scenario**				
	**A**	**B**	**C**	**D**	**E**
**Ethanol**	X	X	X	X	X
**Pellets**		X	X		
**Electricity**	X		X	X	X
**District heating**				X	X

#### Changes in process performance

The ethanol yield and the overall energy demand have been shown to have a major effect on the production cost [10,11,27]. To study the effect of changes in the performance of the ethanol process, the ethanol yield (Y) and the energy demand (Q) of the process were varied for the base case (BC) scenarios, resulting in four new cases for each of the scenarios.

*Y*- The enzyme dosage in SSF is reduced by 50%. As a result the yield is assumed to be reduced to 276 litres per ton dry raw material (6.8% lower than in the base case), and the required SSF residence time is increased from 72 to 96 hours.

*Y*+ Pretreatment is improved so that the sugar losses are reduced by 50%, simulated by adjusting the recovery of the water-insoluble and water-soluble parts of each sugar by the same factor. It was also assumed that a yeast able to grow on both hexose and pentose sugars is employed, and that 60% of the xylose and arabinose present in SSF are converted to ethanol together with the hexoses. All other conditions are the same as in the base case. The overall ethanol yield is increased by 12.1% to 332 L/dry ton.

*Q*- SSF is performed at 12.5% WIS. The ethanol yield, SSF residence time and amounts of yeast and enzymes were maintained. A smaller amount of water is used in the process resulting in a reduced energy demand.

*Y+Q*- This case is a combination of the *Y*+ and *Q*- cases, and was included to study whether the effects of improved yield and reduced energy demand are additive.

## Methods

The process model described was implemented in the commercial flow-sheeting program Aspen Plus (version 2004.1 from Aspen Technology, Inc., Cambridge, MA, USA). Physical property data for biomass components such as cellulose and lignin were taken from the NREL database for biofuel components [33]. Mass and energy balances were solved and, based on the results obtained, a design and estimate of the capital cost of the process equipment were obtained, either using the Icarus Process Evaluator (version 2004.2 from Aspen Technology, Inc.), or based on vendor quotations. Working capital was accounted for according to recommendations in the literature [34]. All costs are in Swedish kronor (SEK) (1 EUR 9.5 SEK). The costs and revenues for the feedstock and the co-products are presented in Table 2. The cost of raw material and the income from selling the solid fuel are based on statistics on current Swedish wood prices provided by the Swedish Energy Agency [35]. Electricity and electricity certificates are traded on the Nordic power exchange market *Nord Pool *[36] where prices vary on a daily basis. It was assumed that the fuel used to generate electricity provided to the grid entitled the producer to certificates. The cost of buying electricity (scenario B) is based on the spot price, but also includes fees for certificates and the grid, as well as taxes. The enzyme cost was assumed to be 19 SEK per million FPU (2.0 EUR/10^6 ^FPU), based on an estimate for producing the enzymes on-site [37]. (The cost corresponds to 0.60 SEK/L ethanol assuming base case yield and dosage.) Other costs used in the study are reported elsewhere [27].

**Table 2:** Costs and prices used in the economic evaluation (1 EUR ≈ 9.5 SEK).

*Costs:*		
Raw material	132 SEK/MWh	(562 SEK/dry ton)
Electricity^*a*^	450 SEK/MWh	
*Ethanol and co-product revenue:*		
Ethanol	924 SEK/MWh	(5.50 SEK/L)
Pellets	195 SEK/MWh	(1146 SEK/dry ton)
Electricity, spot price	350 SEK/MWh	
Electricity certificate	200 SEK/MWh	
District heating	280 SEK/MWh	

^*a*^Only applicable in scenario B

The investment and operating costs and the income from selling the ethanol and the co-products were summed on a yearly basis. The annual capital cost was determined by multiplying the fixed capital investments by an annuity factor of 0.110, corresponding to a depreciation period of 15 years and an interest rate of 7%. A minimum ethanol selling price (MESP) was calculated for each case as a measure of the production cost. In this case, the ethanol price in Table 2 was not used. The MESP is defined as the ethanol selling price resulting in breakeven between the annual costs and the annual income.

## Results and discussion

### Energy demand

The energy demand of the ethanol process and the heat losses that occur have been studied in detail in previous studies [27,38]. The ethanol process includes three energy-demanding steps: steam pretreatment, distillation and evaporation. Preheating the SSF broth also contributes significantly to the overall energy demand. The steam consumption in pretreatment is highly dependent on the moisture content of the raw material, while in distillation and evaporation the feed concentrations (of ethanol and water-soluble non-volatile substances, respectively) have a major effect on the energy demand. The drying step in scenarios B and C requires high-pressure steam, but also generates low-pressure secondary steam that can be utilized in other process steps. Consequently, the net steam demand of the drying step is low.

The process heat duty, i.e. the heat in the form of steam provided to the ethanol process by the steam boiler, varies between 31.6 and 32.3 MW (15.3-15.6 MJ/L ethanol) for the base case scenarios. The *Y*- cases have a slightly lower total heat duty than that in the base cases, while the total heat duty in the *Y*+ cases are somewhat higher. This is due to differences in the energy demand of the distillation and evaporation steps. It should be noted that although the energy demand is higher, the cases with higher ethanol yield result in a lower cost per litre of ethanol. A reduction in the amount of water used in the process decreases the energy demand in distillation and evaporation. As a result, the heat duties of the *Q*- cases are reduced to 26.5-27.0 MW (12.8-13.1 MJ/L ethanol).

While the energy demand of the ethanol production process in the different scenarios (A-E) is similar, there are differences in the utilization of waste heat. Heat losses (defined as unutilized heat streams relative to a state at ambient temperature and water in the liquid phase) occur in the condensers following the rectification column and the last evaporator. In scenario E the heat losses are reduced by utilizing the latent heat leaving the evaporation system for district heating. For utilization of the heat that is removed by cooling in the condenser of the rectification column, a heat pump is required to raise the temperature. Heat pumps were not considered in the current study. The high moisture content of the fuel used in the steam boiler results in a considerable amount of heat leaving the combustor in the form of water vapour in the flue gases. In scenarios D and E the heat losses of the steam generation system are reduced by the implementation of a flue gas condenser. A significant amount of heat is also unutilized in waste water treatment, but as the process step is associated with great uncertainties it will not be further discussed here.

### Energy efficiency

**Figure 2:**
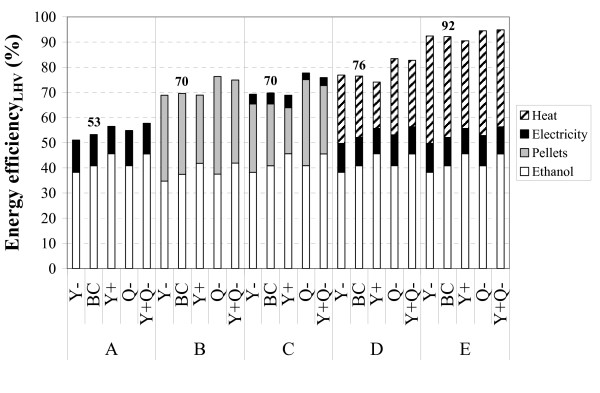
The contribution of each product to the overall energy efficiency.

The energy efficiency can be defined in many ways, which sometimes makes it difficult to compare the results of different studies. The energy efficiency in the current study, as presented in Figure 2, was defined as the energy output in the products (ethanol, pellets, excess electricity and/or heat for district heating) divided by the energy input, comprised of the raw material, a minor contribution from the addition of enzymes and molasses and, in scenario B, the electric power requirement (recalculated as the fuel necessary to produce this electricity, assuming an electricity-to-fuel ratio of 0.4). The calculations were based on the LHVs of the raw material, pellets, ethanol, etc. The energy output is comprised of different energy sources, which will be used for different purposes. For instance, ethanol will be used as a transportation fuel, the pellets for heat and power generation and heat for district heating, while there is a multitude of applications for electricity. Hence, caution should be exercised when making comparisons of the energy efficiency between the different scenarios.

The base case overall energy efficiencies varied from 53% (scenario A) to 92% (scenario E). The major difference was that the heat removed by cooling water in scenario A was utilized for district heating in scenario E. The pellet-producing scenarios B and C are similar in terms of overall energy efficiency. The energy output is higher in scenario B but, as electricity has to be imported from an external source, the energy input is also higher.

Of the 106.1 MW_LHV _originating from the raw material, 43.7 MW_LHV _or 41% ends up as ethanol assuming the base case ethanol yields. In the cases with higher yields the figure is increased to 48.7 MW_LHV _(46%). (Theoretically, i.e. if all carbohydrates in the raw material are converted to ethanol, the ethanol output would be 71.2 MW_LHV_, corresponding to 67% of the lower heating value of the feed-stock.) The effect of changes in the ethanol yield on the overall energy efficiency differs between the scenarios. For scenarios B and C a change in the ethanol yield is compensated for by a change in the amount of pellets produced, resulting in reasonably similar overall energy efficiencies. In the other scenarios, a change in the ethanol yield affects the amount of fuel used in the steam boiler; for example, if the ethanol yield is lower the amount of heat and electricity generated will be higher. For scenarios D and E this results in a higher overall energy efficiency when the ethanol yield is lower, while the opposite result is obtained in scenario A, due to the low degree of utilization of the residues in this case.

A reduction in the energy demand of the ethanol process increases the energy output in the form of co-products. Consequently, the *Q*- cases result in a significant increase in the overall energy efficiency, especially for scenarios B, C and D. The efficiency in scenario E is only slightly higher, as the reduced energy demand results in a lower amount of heat available in the condenser following the last evaporator. The increase of a little more than two percentage units reflects energy savings that occur mainly in the distillation step. Regarding scenario A, the ethanol yield has a greater effect on the efficiency than energy savings in the process, due to the reason mentioned above.

The above results can be summarised as follows:

- The dominant contribution from ethanol to the overall energy efficiency in scenario A makes the efficiency of this scenario sensitive to changes in the ethanol yield.

- For scenarios B, C and D changes in the energy demand have a greater effect on the overall energy efficiency than changes in the ethanol yield.

- The high degree of waste heat utilization in scenario E leaves the overall energy efficiency almost unaffected by changes in yield and energy demand.

### Production cost

**Figure 3:**
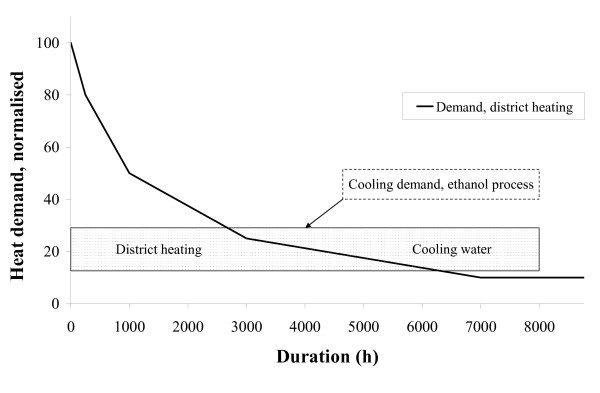
Duration diagram of a fictive but typical Swedish district heating system. The rectangle represents the cooling demand of the ethanol process. The fraction below the curve is used for district heating.

The demand for district heating is highest during the winter and least in the summer. This was taken into account in the economic evaluation. In a typical Swedish district heating system there is a factor of roughly ten between the minimum and maximum heat demand. In scenario E, 42.9 MW heat is produced. For this to be delivered all-year round, a system with a maximum capacity of 430 MW and an annual delivery of approximately 1000 GWh is required. There are currently less than 10 systems of this size in Sweden. Scenario D, with a heat production of 26.1 MW, may be somewhat easier to implement, as there are around 30 Swedish district heating systems of adequate size. The most likely scenario is that a district heating system will be provided with heat from the plant during part of the year. In the economic evaluation it was assumed that heat is delivered to a district heating system during a period of time equivalent to 4500 hours of maximum capacity annually. Cooling water is used during the remaining 3500 hours to remove the heat, i.e. the process operation is similar to scenario A during this period. An example of how heat from the ethanol plant can be delivered to a district heating system, according to the assumptions discussed above, is shown in Figure 3.

#### Annual cash flows

**Figure 4:**
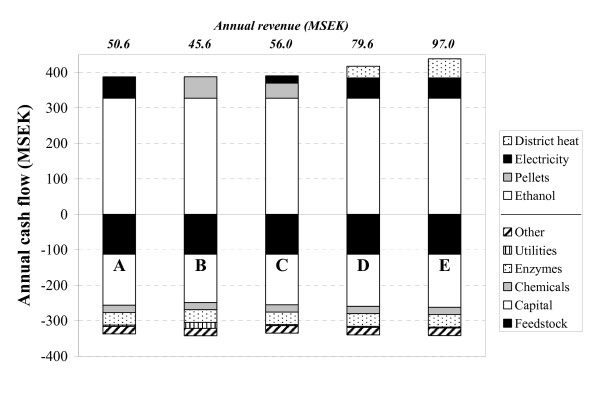
Annual costs and revenues for scenarios A-E. "Other" includes the cost of labour, maintenance and insurance. For scenarios D and E it was assumed that there is an annual demand for district heating during a period of time equivalent to 4500 hours of full capacity. During the remaining 3500 hours the heat is removed with cooling water and hence does not generate any income.

The annual income and costs associated with each scenario are presented in Figure 4. The main costs are those for feedstock and capital. The estimated investment cost ranges between 1200 (scenario B) and 1340 million SEK (scenario E) (126-141 MEUR), with the pretreatment and steam generation systems, together with the SSF fermentors, being responsible for the major contributions to the cost. There are only small differences in the total cost between the scenarios. Scenario B, with the lowest capital cost, has the highest total annual running cost (342.1 MSEK, 36.0 MEUR), due to the higher utility cost as electricity has to be purchased in this case. The main difference between the scenarios lies in the annual income from selling the co-products. This figure varies from 59.9 MSEK in scenario A to 110.9 MSEK in scenario E (6.3-11.7 MEUR). However, the income from selling the ethanol (327.6 MSEK, 34.5 MEUR) is much higher, and, although the co-product revenue is important for the overall economics, Figure 4 clearly shows that ethanol is the main product.

#### Minimum ethanol selling price

**Figure 5:**
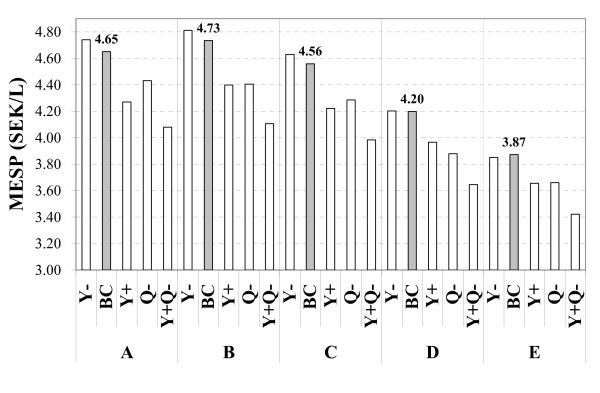
Minimum ethanol selling price (MESP) for the proposed scenarios.

The estimated MESP for each case is presented in Figure 5. For the base cases the MESP varied between 3.87 (scenario E) and 4.73 SEK/L ethanol (scenario B) (0.41-0.50 EUR/L). This variation is due to different ways of utilizing the residue streams. The income from selling the electricity certificates makes it beneficial to generate electricity. As the revenue for electricity is higher than for pellets, the MESP of scenario A is lower than in scenario B. Scenario C with a similar energy efficiency to scenario B (see Figure 2), but with some of the output in the form of electricity, results in a lower MESP (4.56 SEK/L, 0.48 EUR/L). At an electricity spot price of 429 SEK/MWh (45 EUR/MWh) (with the certificate price maintained at 200 SEK/MWh) the same MESP (4.51 SEK/L) is obtained for scenarios A and C. Without the extra revenue of 200 SEK/MWh_el _from selling the electricity certificates, the base case MESP of scenario A would be 0.36 SEK/L higher. The increase in scenarios D and E would be 0.34 SEK/L, and for scenario C 0.12 SEK/L. The MESP of scenario B remains unaffected as no electricity is generated.

The scenarios with district heating (D and E) are by far the most profitable alternatives. Compared to scenario C, the MESP is 0.36 (scenario D) and 0.69 (scenario E) SEK lower per litre of ethanol. District heating revenues of 97 and 68 SEK/MWh for scenarios D and E, respectively, give an identical MESP to that in scenario C, i.e. a considerably lower price than the 280 SEK/MWh assumed in this study. With an 8000-hour delivery of district heating, the MESP of scenarios D and E would be 3.79 and 3.18 SEK/L (0.40/0.33 EUR/L), respectively. However, finding a location where this could be implemented would be difficult.

#### Effect of variations in ethanol yield and/or energy demand

The cases with lower ethanol yield result in a lower total annual cost, mainly due to the reduced enzyme cost, and a higher co-product revenue compared with the base cases, which to some extent compensates for the smaller amount of ethanol produced. Accordingly, the higher the impact of co-product revenue on the economics, the lower the negative effect of a reduced yield. In scenario E the *Y*-case actually results in a lower MESP than in the base case. However, with the assumed ethanol selling price, the annual revenue decreases with a lower yield. A higher yield is beneficial for the overall economics, although it results in reduced co-product revenue, which affects scenarios D and E to a greater extent. In the *Y*+ cases the MESP is reduced by 0.38 SEK/L in scenario A, but only by 0.22 SEK/L in scenario E compared with the base cases.

The results presented in Figure 5 clearly show that the ethanol yield has a high impact on the process economics, which makes it a key factor for the successful full-scale introduction of ethanol production from lignocellulosic feedstock. The yield dependency is reduced in scenarios D and E, which may make the implementation of these scenarios economically less risky.

As a consequence of the reduced energy demand, the *Q*-cases result in lower MESP for all scenarios due to lower investment costs and higher co-product revenue. The effect on the production cost of a reduced energy demand is higher for scenarios B and D. For scenarios A and E a higher WIS concentration does not affect the production cost as much, due to the same reasons as mentioned regarding the effect on the energy efficiency. In scenario C, the reduced demand for steam results in lower electricity production which, to some extent cancels out the positive economic effects of a more energy-efficient process. For a plant configured according to scenario C, a further reduction in steam demand will, at a certain point, result in a need for electricity import.

In scenarios A, B and C the *Y+Q*- case results in a reduction in MESP almost equal to the sum of the reductions obtained for the *Y*+ case and the *Q*- case. In other words, the positive effects on the economics of a higher yield and a lower energy demand are almost additive. In scenarios D and E the reduction of MESP in the *Y+Q*- case actually exceeds the sum of the reductions of the *Y*+ and the *Q*-cases. The reason for this lies in the process conditions set, in particular keeping the inlet and outlet temperatures of the flue gases in the flue gas condenser fixed at 150 and 50°C in all cases, and the fact that a large proportion of the steam generated in the steam boiler was withdrawn for use in the ethanol process. As a result, the entire flue gas stream could not be utilized in the flue gas condenser in the base cases and *Y*+ cases, as this would result in a temperature crossover (the constraint was set at a minimum temperature difference anywhere within the flue gas condenser of 1°C). For the other cases this was not a problem. Consequently, the amount of heat available for district heating was somewhat reduced in the base cases and *Y*+ cases in comparison with the other cases.

## Conclusion

This study clearly shows the importance of good utilization of the residue streams and the impact this has on the process economics. With the current selling prices of the co-products (pellets, electricity and heat for district heating), utilization of the excess solid residue for heat and power production is highly favourable from an economic point of view. The introduction of electricity certificates promotes electricity generation. The implementation of district heating enables the utilization of streams present at temperatures that are difficult to make use of by other means, but also limits the plant location options as there must be a demand for the heat available. If excess heat cannot be delivered to a district heating system, scenario C, with back-pressure power generation and pellet production from the excess solid residue, is the most favourable alternative. This is also the case without the income from selling the electricity certificates.

This study has also shown that an improvement in the ethanol yield and reduction in the energy demand have a strong positive effect on the process economics, almost independently of each other. The design of the ethanol production process was very similar for all scenarios, as the aim of this study was to illustrate the impact on the production cost of co-product utilization. It should be mentioned that the conclusions drawn here may change when considering other configurations of the ethanol production process. For instance, increasing the number of evaporators from five to eight, or implementing mechanical vapour recompression to evaporation, have both been shown to reduce the production cost in previous studies [11,38]. However, the former alternative cannot be applied to scenario E, the economically most favourable scenario in this study, as it would result in unrealistically small temperature differences between the evaporators. In the latter alternative the electric power requirement will increase, which must be weighed against the reduction in steam demand. Finally, replacing evaporation with anaerobic digestion, as suggested by Wingren et al. [11], would significantly reduce the overall energy demand. It would also have a considerable effect on the amounts of pellets, heat and power produced, if the biogas produced is upgraded for use as vehicle fuel instead of being burnt on-site. This configuration seems promising, but was not considered here, as there are considerable uncertainties regarding the performance of anaerobic treatment of the stillage stream from a wood-to-ethanol process and the treatment of the resulting sludge.

## Abbreviations

*COD*: Carbon oxygen demand; *DM*: Dry matter, i.e. the total amount of non-volatile substances; *EUR*: Euro (currency used in European Monetary Union); *FPU*: Filter paper units, which are used as a measure of the enzyme activity; *LHV*: Lower heating value, i.e. the heat released in combustion relative to a reference state of 25°C and water in vapour phase; *MESP*: Minimum ethanol selling price, defined as the ethanol sales price resulting in a breakeven between the annual costs and the annual income; *SEK*: Swedish kronor (1 EUR 9.5 SEK); *SSF*: Simultaneous saccharification and fermentation; *WIS*: Water-insoluble solids, or water-insoluble non-volatile substances.

## Competing interests

The authors declare that they have no competing interests.

## Authors' contributions

Per Sassner constructed the Aspen Plus process model, carried out the process simulations and economic evaluation, analysed the results and wrote the paper. Guido Zacchi conceived of the study, and participated in its design and coordination and helped to draft the manuscript. All authors read and approved the final manuscript.
